# Hoof Health in the Parmigiano Reggiano Dairy Production System: A Pilot Epidemiological Study Using Digitally Recorded Hoof-Trimming Data

**DOI:** 10.3390/ani16203195

**Published:** 2026-10-12

**Authors:** Maria Virginia Ralletti, Fabio Testori, Luigi Grasselli, Raffaella Finocchiaro, Lorenzo Benzoni, Maurizio Marusi, Alessandro Spadari

**Affiliations:** 1 Department of Veterinary Medical Sciences, University of Bologna, 40064 Ozzano dell’Emilia, Italy; 2 Independent Researcher, 20081 Abbiategrasso, Italy; 3 Independent Researcher, 42100 Reggio Emilia, Italy; 4 Associazione Nazionale Allevatori della Razza Frisona Bruna e Jersey Italiana, 26100 Cremona, Italy

**Keywords:** dairy cattle, claw lesions, hoof health, hoof trimming, digital recording, risk factors, Parmigiano Reggiano

## Abstract

The aim of this study was to describe the prevalence and distribution of claw lesions in dairy cows from farms producing milk for Parmigiano Reggiano cheese and to evaluate factors associated with their occurrence. Data were collected during hoof-trimming visits performed by four professional hoof trimmers on 25 farms between March 2023 and October 2024 using a specifically developed digital recording application. 4410 visits from 3066 cows were included. At least one lesion was recorded in 53.5% of visits and 44.3% of cows. Sole ulcer was the most common lesion (23.8% of visits), followed by sole haemorrhage (19.3%), wall abscess (18.1%), dermatitis (16.4%), and wall lesions (10.3%). Mechanically related lesions mainly involved the lateral claws of the hind limbs. Parity was strongly associated with lesion occurrence: compared with first-parity cows, odds increased at parity 2 (OR 1.41), parity 3 (OR 2.52), and parity ≥4 (OR 3.14). Because parity and age were highly correlated (r = 0.94), this association should be interpreted as a combined parity/age effect, and their independent effects could not be distinguished. Lactation stage showed weaker associations, and seasonal and farm-level differences were observed. Overall, non-infectious lesions predominated. The marked variation among farms suggests that herd-level factors contribute to claw health. Linking these digitally recorded hoof-trimming data with national breeding and production databases may support large-scale surveillance and future epidemiological and genetic investigations.

## Simple Summary

This study investigated claw lesions in dairy cows from farms producing milk for Parmigiano Reggiano cheese, a production system characterized by specific feeding and management requirements. Data were digitally recorded by professional hoof trimmers during trimming. A total of 4410 visits involving 3066 cows from 25 farms were analyzed. Claw lesions were recorded in 53.5% of visits and affected 44.3% of cows during the study period. Sole ulcer, sole hemorrhage, and wall abscess were the most frequently recorded lesions, and mechanically associated lesions occurred mainly in the hind lateral claws. Claw lesions were more common in cows of higher parity/older age. Since age and parity are closely related, their individual effects could not be separated, while lactation stage showed weaker associations. Considerable differences were also observed among farms, suggesting that herd-level conditions may contribute to claw health. These findings show that routinely collected digital hoof-trimming records can provide valuable information for monitoring claw health and may contribute to large-scale surveillance and prevention programs among Italian dairy cattle population.

## 1. Introduction

Lameness in dairy cattle is a major animal welfare concern [1] and over 90% of lameness cases are associated with foot disorders [2,3,4,5], although foot lesions do not necessarily result in lameness [6]. Lameness negatively affects longevity [7], productivity [8], and reproductive performance [9,10]. As a result, it is considered one of the most economically impactful disorders in the dairy industry [11]. The prevalence of claw disorders is influenced by a range of risk factors, including environmental conditions, housing design, hygiene and management practices and genetics [12,13,14].

Claw lesions are generally classified as either infectious—such as digital dermatitis, interdigital dermatitis, heel erosion, and interdigital phlegmon—or non-infectious, including white line disease, sole ulcer, and sole haemorrhage [14,15]. Infectious lesions are primarily associated with environmental hygiene, whereas non-infectious lesions are typically linked to metabolic and/or mechanical factors [15,16,17]. Digital dermatitis, sole haemorrhage, sole ulcer, and white line disease are consistently reported as the main causes of lameness in dairy cattle [17,18,19].

Routine hoof trimming represents a widely recognized effective tool for the prevention and management of claw disorders and allows the collection of data on claw health in a large number of cows accurately reflecting the true status of hoof health in a population. Additionally, it can increase the farm profitability [20] and permits the systematic collection of claw health enabling monitoring and analysis of lesion presence and absence at the individual cow, group, and herd levels. In several countries, digital recording systems have been developed, most of which are based on data collected by professional hoof trimmers [21].

Although the prevalence of lameness has been widely investigated in several countries, data on the epidemiology of claw lesions in Italy remain scarce. This lack of information is particularly relevant when considering specific dairy production systems that may differ in feeding and management practices. The Parmigiano Reggiano production system is a Protected Designation of Origin (PDO) dairy production chain located in Northern Italy, encompassing the provinces of Parma, Reggio Emilia, Modena, and parts of Bologna and Mantua. Milk production is regulated by specific requirements regarding geographical origin, feeding management, and farming practices. These production rules distinguish this system from conventional dairy production and represent one of the most important examples of quality-oriented dairy production in Europe, playing a significant role in the rural economy and landscape conservation of the region [22]. In particular, feeding regulations include the prohibition of silage and the use of local forage-based diets. These nutritional requirements were established primarily to preserve cheese quality, but they may also be relevant to claw health through their potential influence on rumen conditions and the risk of subacute ruminal acidosis, a recognized predisposing factor for claw horn lesions [23]. The aim of this study was to describe the prevalence and distribution of claw lesions recorded during hoof-trimming visits in dairy herds producing milk for Parmigiano Reggiano cheese and to evaluate factors potentially associated with their occurrence.

## 2. Materials and Methods

**Figure 1:**
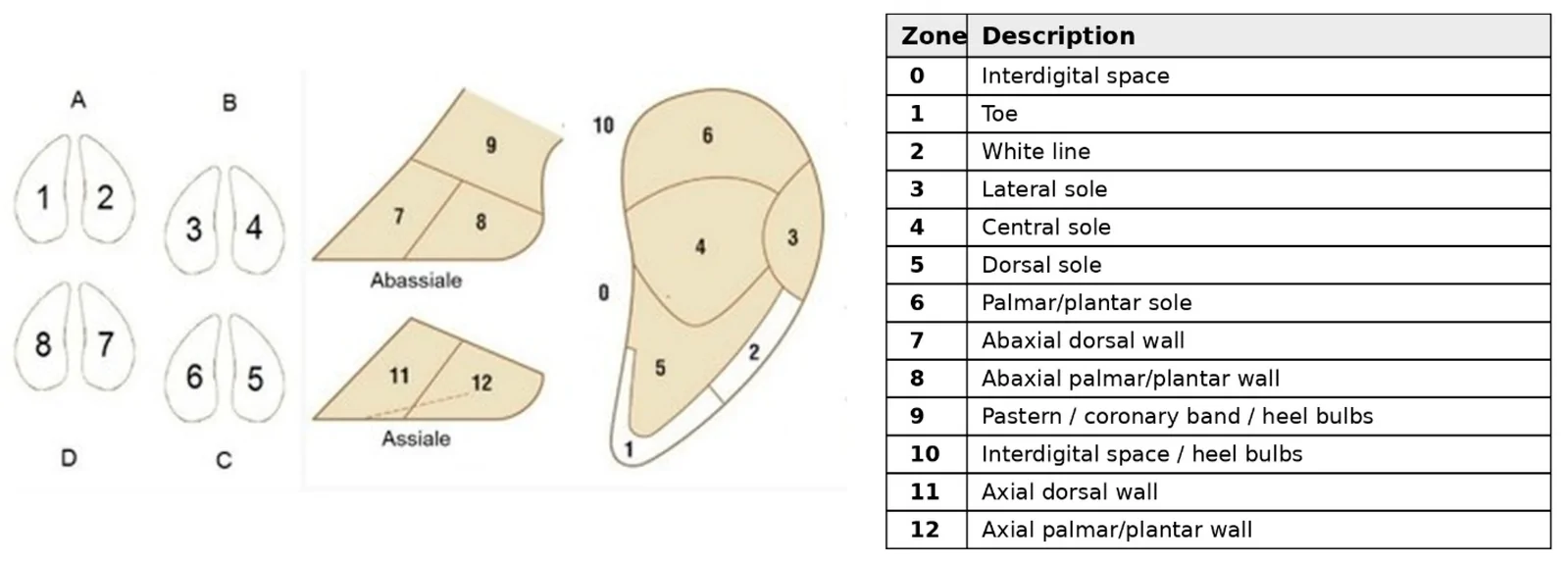
Interface of the digital recording application used by hoof trimmers during trimming visits. The operator selects the affected foot (A: front left, B: front right, C: hind right, D: hind left), claw (1: front left lateral (1-FL-L), 2: front left medial (2-FL-M), 3: front right medial (3-FR-M), 4: front right lateral (4-FR-L), 5: hind right lateral (5-HR-L), 6: hind right medial (6-HR-M), 7: hind left medial (7-HL-M), 8: hind left lateral (8-HL-L)) and, subsequently, anatomical zone on the claw diagram and the type of lesion observed. Definition of anatomical zones by numbers 0–12 is included in the figure. Foot lesion identification map used was developed by International Lameness Committee (2008) in cooperation with Zinpro Corporation (Eden Prairie, MN, USA). Figure courtesy of Zinpro Corporation (Eden Prairie, MN).

Data were collected between March 2023 and October 2024 as part of routine and on-demand hoof trimming visits performed by four professional hoof trimmers in 25 Holstein dairy herds producing milk for Parmigiano Reggiano cheese. The distribution of observations across hoof trimmers is reported in Supplementary Table S1. No formal inter-observer calibration or agreement assessment was performed as part of the study. General information (visit date, farm and cow identification number) and lesion-specific data were recorded during the trimming. The data were collected using a computerized recording system named Datatrim (FAMA Services S.R.L.S., Abbiategrasso, Italy) installed on a tablet that trimmers use in their daily work routine. Each trimming session generated one record per lesion observed, including the affected claw (foot quadrant and laterality), the anatomical zone within the claw, the lesion type and cow-level information (cow identification number, parity, days in milk, cumulative milk yield, date of birth, farm identifier). Lesion data included type of lesion and lesion location by foot, claw and area of onset (Figure 1). When the same lesion type was recorded in more than one anatomical zone of the same claw during the same visit, these annotations were consolidated and counted as a single lesion record.

Cow-level information was automatically linked to the dataset through the national animal identification number, allowing pedigree and milk-production data to be retrieved from the central database of the Italian National Association of Holstein, Brown Swiss and Jersey Breeders (ANAFIBJ).

### 2.1. Dataset Construction

The raw extraction from the Datatrim application contained one row for each event recorded during a visit: one row per lesion when one or more lesions were observed, and a single row (coded “#NA#” in the lesion-type field) when the trimmer recorded no claw lesion during that visit. Because visits with multiple lesions generated multiple rows sharing the same animal, date and trimmer combination, a visit-level identifier was constructed by combining animal identity, visit date, and trimmer code, and rows belonging to the same visit were transformed into a single record. Two categories present in the raw data, “Other” (OTH) and “Non-Claw Lameness” (NCL), were excluded prior to analysis: OTH annotations originated almost exclusively (99.8%) from a single trimmer, indicating a data-entry artefact rather than a genuine clinical finding. NCL records non-claw lameness and was therefore outside the scope of the present study. Visits in which OTH/NCL were the only recorded entries (*n* = 16) were treated as missing rather than lesion-free. Visits in which the only entry was #NA# were retained as lesion-free records, allowing prevalence to be estimated against a true at-risk denominator rather than only among already-affected animals. The final dataset comprised 4410 visits from 3066 animals.

Lesion types with at least 100 recorded occurrences were retained as individual outcomes and modelled separately: Sole Ulcer (SU), Sole Hemorrhage (SH), Wall Abscess (WA), Dermatitis (D), Wall Lesion (WL), Thin Sole (TS), Apical Lesion (AL), Corkscrew Claw (CC), and Phlegmon (P). Rarer lesion types like Tyloma (T), Heel Erosion (HE), Laminitis (L) (combined *n* = 45) were retained for descriptive purposes only and excluded from multivariable modelling due to insufficient statistical power. A complete mapping of the original Datatrim lesion codes, the categories used in the present study, and their correspondence with standard claw-lesion terminology is provided in Supplementary Table S2. For the present analysis, the Digital Dermatitis (DD), Interdigital Dermatitis (DI), and non-specific dermatitis records were consolidated into a single Dermatitis (D) outcome. For each visit, presence/absence of each lesion type was coded as a binary variable, since multiple lesion types could co-occur within the same visit.

### 2.2. Statistical Analysis

All analyses were performed in Python 3.11.15. Data handling was performed using pandas 3.0.2, numerical and statistical computations using NumPy 2.4.4 and SciPy 1.17.1, and graphical outputs using Matplotlib 3.10.9. Logistic regression and mixed-effects models were fitted using custom NumPy/SciPy routines. Visit-level prevalence was calculated as the proportion of hoof-trimming visits with at least one recorded claw lesion among all included visits, whereas animal-level prevalence was calculated as the proportion of cows with at least one recorded lesion during the study period among all cows included in the dataset.

Co-occurrence between lesion types within the same visit was assessed using the phi coefficient. The anatomical distribution of each lesion type was examined by cross-tabulating lesion type against claw position (eight-claw coding by front/hind, left/right, lateral/medial) and foot zone.

Risk factors for lesion occurrence were assessed using multivariable logistic regression, fitted by maximum likelihood, with parity (categorized as 1, 2, 3, ≥4), lactation stage (0–100, 101–200, 201–305, 306–370, >370 days in milk), and season of visit as fixed effects. Standard errors were clustered at the animal level (sandwich/robust variance estimator) to account for repeated visits on the same cow. The presence of at least one claw lesion was considered the primary outcome. The same multivariable model was subsequently fitted separately for each of the 9 individual lesion types as secondary exploratory analyses. In all models, the same set of fixed effects (parity, lactation stage, and season) and the same animal-clustered robust variance estimator were used. To account for multiple testing in the lesion-specific analyses, the Benjamini–Hochberg false discovery rate (FDR) correction was applied jointly to the 90 coefficient-level *p*-values from these nine models (9 lesion types × 10 coefficients, excluding the intercept). FDR-adjusted *p*-values (q-values) <0.05 were considered statistically significant. Odds ratios and 95% confidence intervals were used as the main measures of association.

Cumulative milk yield was examined separately rather than jointly with lactation stage due to substantial collinearity between the two variables (r = 0.65–0.67). For descriptive purposes, visits were ranked according to cumulative milk yield and divided into quintiles (Q1–Q5, approximately 20% of visits each). Because cumulative milk yield is strongly dependent on DIM and parity, repeated visits from the same cow could contribute to different quintiles, and milk production may itself be affected by claw lesions. Therefore, cumulative milk yield was examined only as an exploratory descriptive variable and was not included in the risk-factor models or subjected to inferential testing. A high correlation (r = 0.94) between parity and animal age precluded the inclusion of both variables in the same model; the parity effect is therefore reported as a combined parity/age effect rather than attributed to parity alone.

As a sensitivity analysis, we refitted the multivariable model for the presence of at least one claw lesion accounting for farm in two different ways: first as a fixed effect (24 indicator variables) and then as a random intercept. The latter model was fitted by maximum likelihood using adaptive Gauss–Hermite quadrature. This allowed us to assess whether the associations with parity, season, and lactation stage were affected by farm-level clustering. We also attempted a fully nested model with cow within farm, but it did not provide stable estimates, likely because 73% of cows had only one recorded visit. The nested model was therefore not retained.

Additional sensitivity analyses were performed to examine the robustness of the temporal associations. First, calendar year (2023 or 2024) was added to the main multivariable model. Second, season was replaced by calendar month to examine temporal variation at a finer scale, and model fit was compared using the Akaike information criterion (AIC). The analysis was then repeated for visits performed during the five months represented in both study years (March and July–October). Standard errors were clustered at the animal level as in the main analysis.

Because trimmers did not share farms, trimmer and farm effects could not be estimated independently. To explore whether the observed between-farm variation could be explained solely by differences among trimmers, raw lesion prevalence by farm was also examined after restricting the dataset to visits performed by the dominant trimmer, who accounted for 80.5% of all visits and attended 22 of the 25 farms. Farm-specific random effects were estimated using empirical Bayes predictions to describe between-farm heterogeneity while accounting for the unequal number of observations across farms.

Full model specifications and implementation details, including the cluster-robust variance estimator, farm random-intercept model, temporal sensitivity analyses, and multiple-testing correction, are provided in Supplementary Methods S1.

## 3. Results

### 3.1. Descriptive Epidemiology

A total of 4410 visits from 3066 animals across 25 farms were included in the analysis. At the visit level, 2361 visits (53.5%) recorded at least one lesion, while 2049 (46.5%) were lesion-free. At the animal level, considering the whole observation period, 1358 animals (44.3%) presented at least one lesion, while 1708 (55.7%) remained lesion-free throughout follow-up. Data collection combined scheduled routine trimming visits (*n* = 3937; 89.3%) with a smaller proportion of on-demand visits (*n* = 473; 10.7%).

Sole ulcer was the most frequent lesion type, recorded in 1050 visits (23.8%) and affecting 523 animals (17.1% of the population), followed by sole hemorrhage (19.3% of visits, 14.0% of animals), wall abscess (18.1%, 14.0%), dermatitis (including digital dermatitis; 16.4%, 12.0%), and wall lesion (10.3%, 7.1%). Thin sole, apical lesion, corkscrew claw, and phlegmon were each present in fewer than 5% of visits (Table 1).

**Figure 2:**
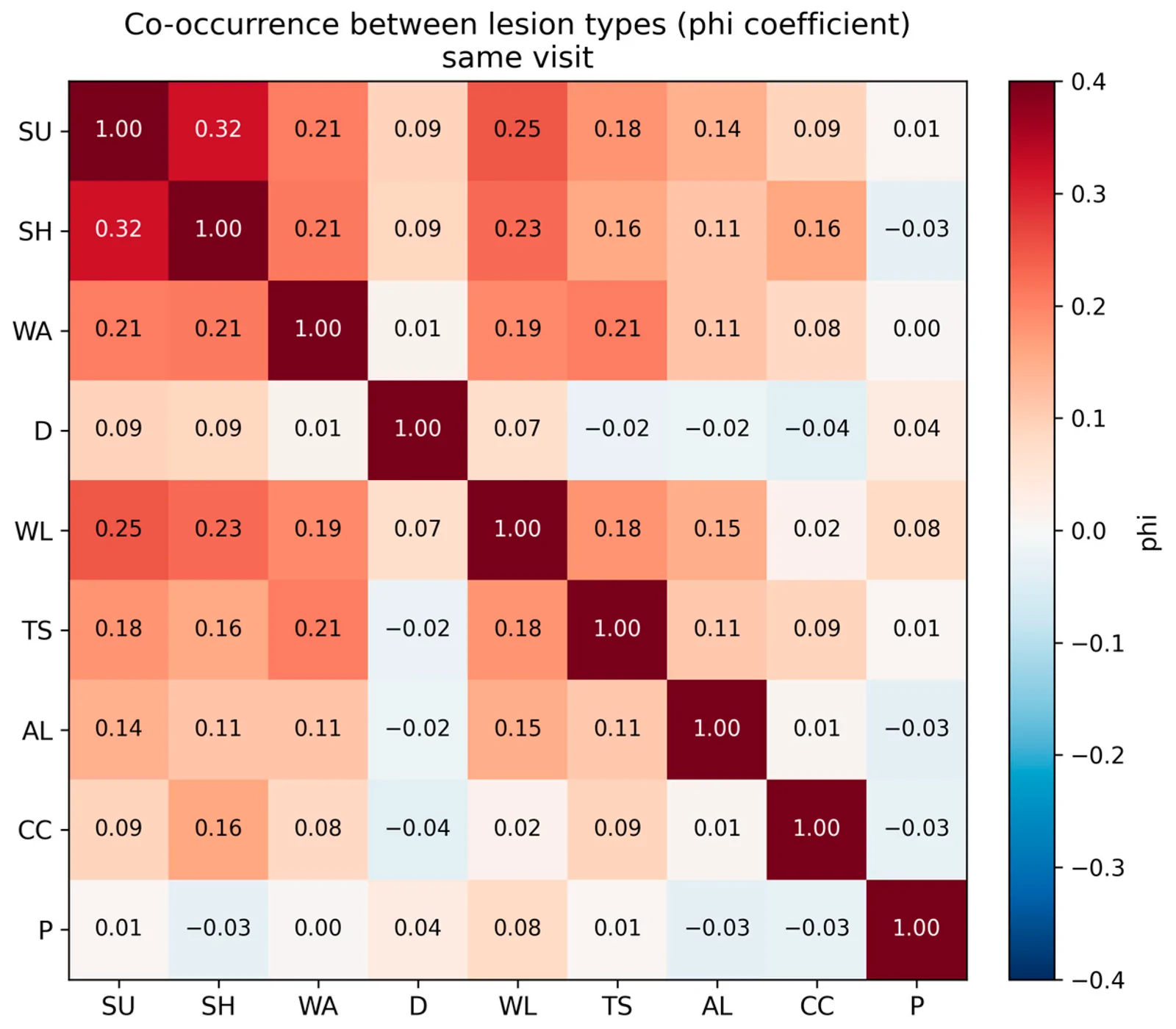
Pairwise co-occurrence of claw lesion types recorded within the same hoof-trimming visit, expressed as phi coefficients. Higher positive values indicate a greater tendency for two lesion types to occur during the same visit.

Positive associations were observed among SU, SH, WA, and WL, with phi coefficients ranging from 0.19 to 0.32. Dermatitis was essentially uncorrelated with the other lesion types (phi ≤ 0.09) (Figure 2). Of the 722 visits classified within this category, 648 (89.8%) included at least one DD record, 38 (5.3%) at least one DI record, and 45 (6.2%) only a non-specific D record; nine visits included both DD and DI.

**Figure 3:**
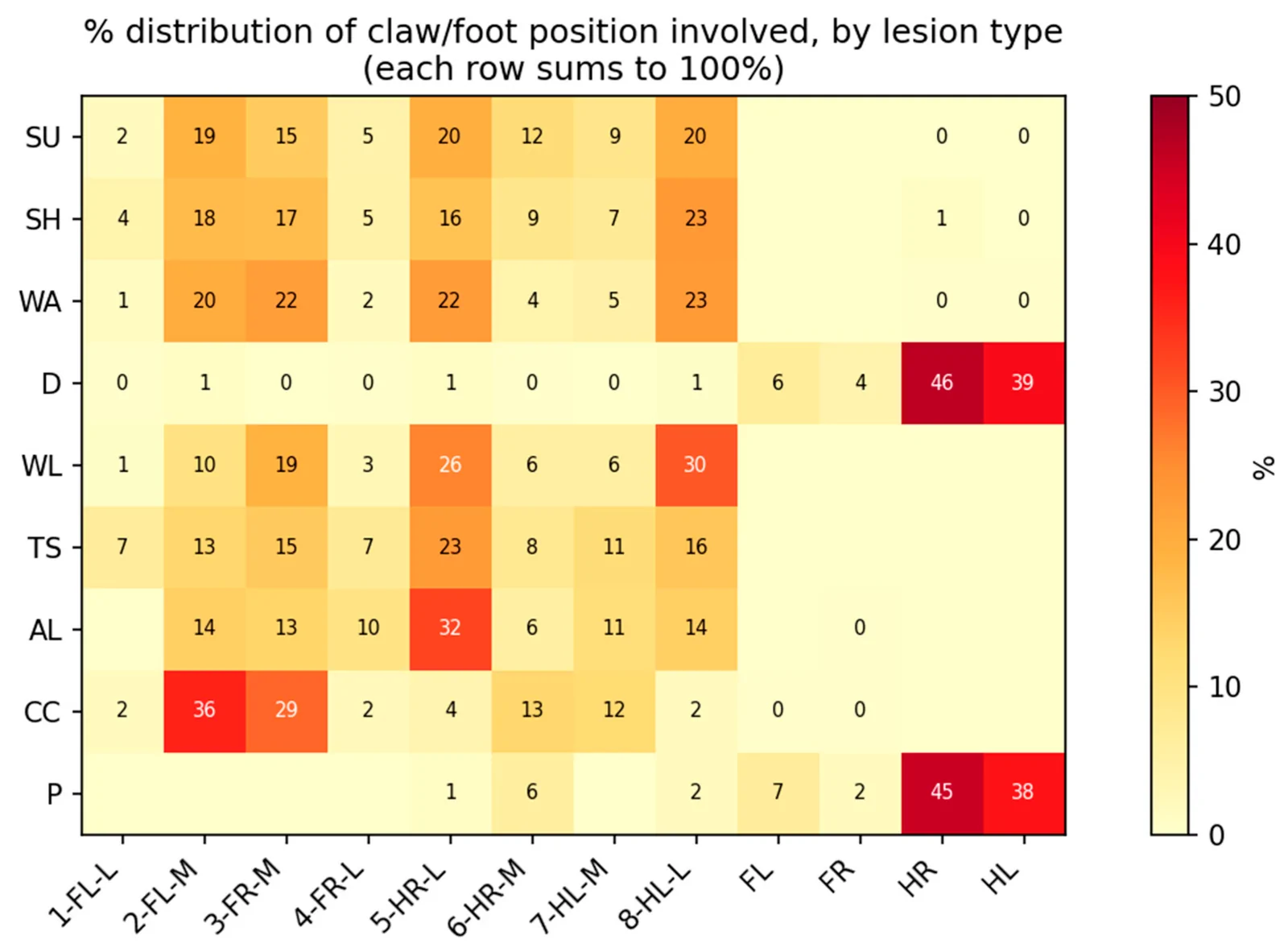
Anatomical distribution of recorded claw lesions according to claw position and affected anatomical zone. Values represent the proportion of observations for each lesion type.

**Figure 4:**
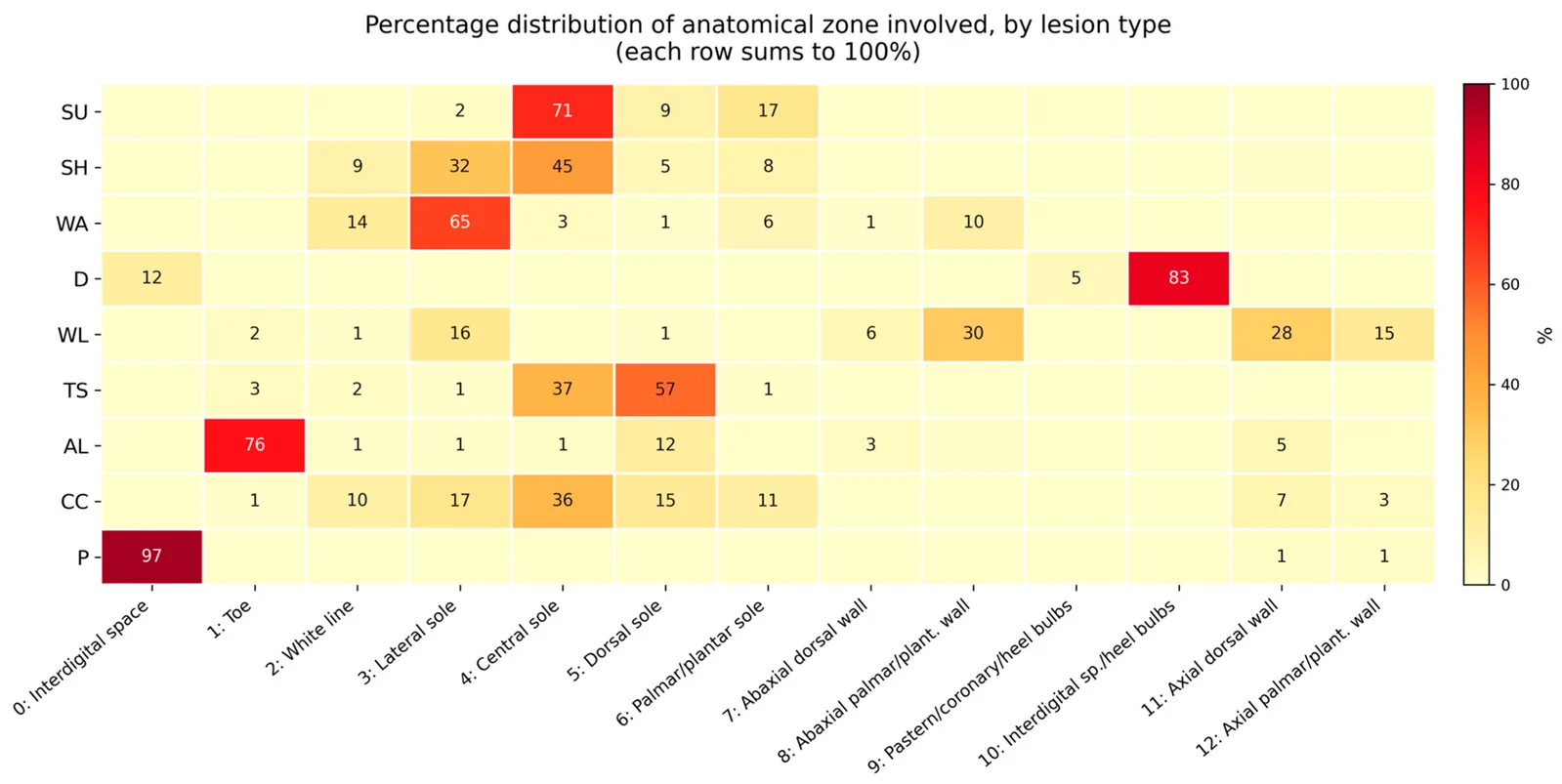
Percentage distribution of anatomical zone involved (zones 0–12, ICAR Claw Health Atlas numbering), by lesion type. Each row sums to 100%: records coded to more than one zone (e.g., “4–5”) were split proportionally across the zones listed.

Lesions of mechanical origin (SU, SH, WA, WL) were predominantly located on the hind lateral claws in positions 5-Hind Right Lateral (5-HRL) and 8-Hind Left Lateral (8-HLL). Corkscrew claw showed the opposite pattern, occurring mainly on the front medial claws, specifically 65% of cases combined across 2-Front Left Medial (2-FLM) and 3-Front Right Medial (3-FRM) (Figure 3). Dermatitis was predominantly recorded in zone 10 (interdigital space/heel bulbs; 83% of coded records), whereas apical lesions were mainly located in zone 1 (toe; 76%) (Figure 4).

### 3.2. Risk Factors for Lesion Occurrence

In the multivariable logistic model (any lesion vs. none, animal-clustered robust standard errors), parity showed the strongest association with lesion occurrence. Compared with primiparous cows, the odds of presenting a lesion were 1.41 times higher at parity 2 (95% CI 1.15–1.71, *p* = 0.001), 2.52 times higher at parity 3 (95% CI 2.01–3.15, *p* < 0.001), and 3.14 times higher at parity ≥4 (95% CI 2.50–3.94, *p* < 0.001).

Because animal age and parity were highly correlated in this dataset (r = 0.94, corresponding to a variance inflation factor of approximately 8), it was not possible to include both variables in the same model; the parity effect reported here should therefore be interpreted as a combined parity/age effect rather than attributable to parity alone.

Lactation stage showed a comparatively modest association with lesion occurrence, with a moderate increase in odds beyond 200 days in milk (OR 1.24–1.29, *p* ≤ 0.025), except for the >370-day category, which did not reach significance (OR 1.23, *p* = 0.120). In the main model season was also associated with lesion risk, with lower odds in spring (OR 0.62, *p* < 0.001) and autumn (OR 0.82, *p* = 0.011) relative to summer, and higher odds in winter (OR 1.46, *p* = 0.001) (Table 2). Monthly lesion prevalence varied considerably, ranging from 30.2% in March to 73.6% in August. Substantial differences were also observed within the same season, particularly between July (41.7%) and August (73.6%) and between September (59.7%) and October (31.1%). The number of visits was unevenly distributed across months, ranging from 103 in January to 953 in September (Table 3). The temporal associations were partly affected by adjustment for calendar year (Supplementary Table S3). After year was included in the model, winter was no longer significantly different from summer (OR 1.09, 95% CI 0.87–1.37), whereas lower odds remained in autumn and spring. Lesion occurrence also differed between study years, with lower odds in 2024 than in 2023 (OR 0.54, 95% CI 0.45–0.64). A model using calendar month instead of season showed marked variation between individual months and provided a better fit than the season-based model (AIC 5587.8 vs. 5814.9) (Supplementary Table S4). When the analysis was restricted to the five months represented in both years, the difference between years remained (OR 0.33, 95% CI 0.26–0.42); spring remained lower than summer, whereas the difference between autumn and summer was no longer significant (Supplementary Table S5).

### 3.3. Lesion-Specific Risk Factor Patterns

**Figure 5:**
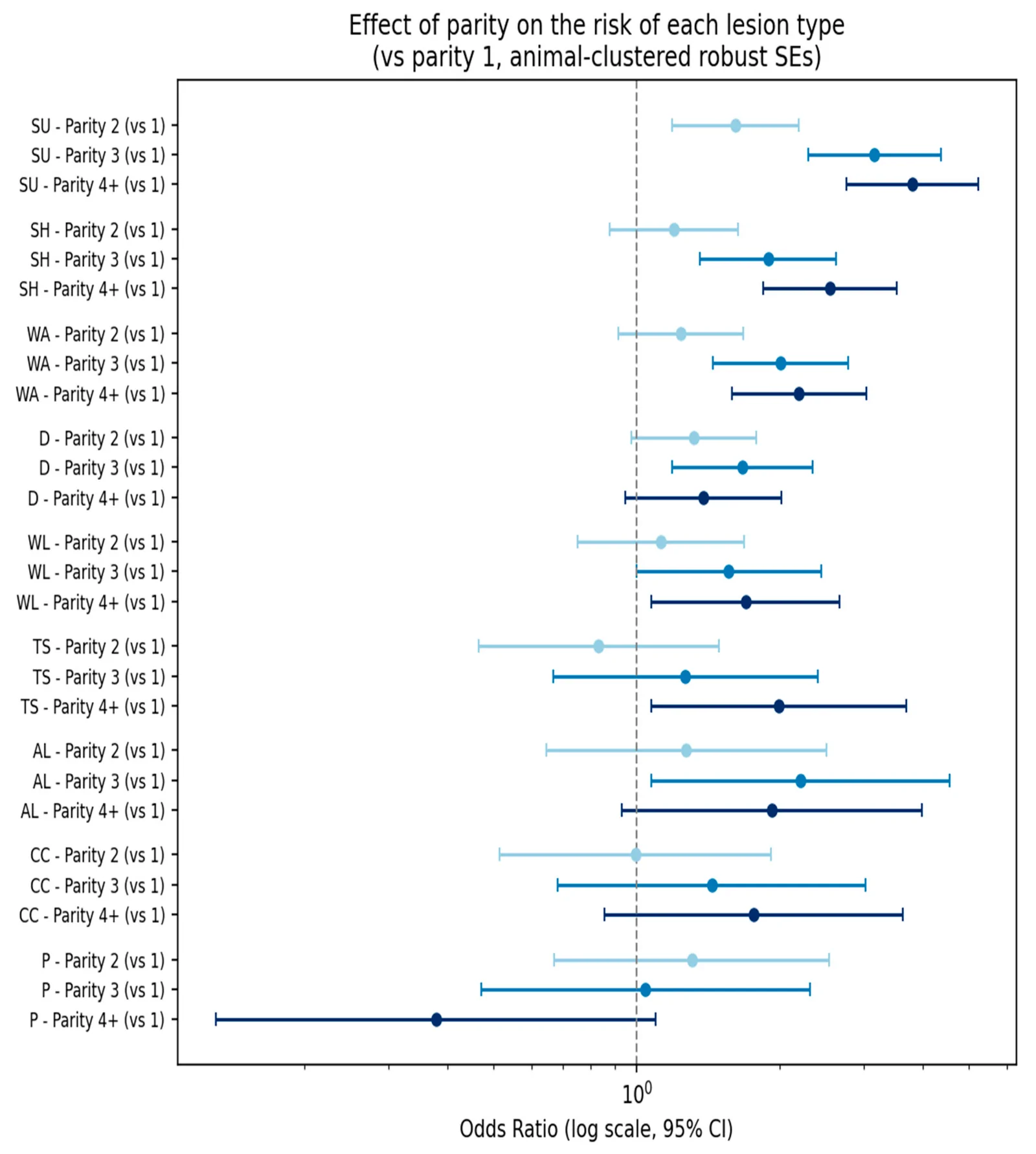
Association between parity and individual claw lesion types estimated using multivariable logistic regression. Odds ratios (OR) and 95% confidence intervals are shown relative to primiparous cows (parity 1), after adjustment for lactation stage and season. Standard errors were clustered at the animal level. Light blue, medium blue, and dark blue indicate parity 2, parity 3, and parity ≥4, respectively. The dashed vertical line marks OR = 1 (no association), and the x-axis is on a logarithmic scale.

The strength of the parity effect varied considerably across lesion types (Figure 5). The strongest associations were observed for sole ulcer (SU), with ORs increasing from 1.61 (95% CI 1.19–2.19) at parity 2 to 3.80 (95% CI 2.77–5.22) at parity ≥4. Higher odds were also observed for sole hemorrhage (SH: OR = 2.55, 95% CI 1.84–3.53 at parity ≥4) and wall abscess (WA: OR = 2.19, 95% CI 1.58–3.04 at parity ≥4), and these associations remained significant after FDR correction. For dermatitis, higher odds were observed at parity 3 (OR = 1.67, 95% CI 1.19–2.35), but not at parity ≥4. Associations previously observed for wall lesion and thin sole at parity ≥4 did not remain significant after FDR correction. No clear association with parity was observed for apical lesion, corkscrew claw, or phlegmon.

**Figure 6:**
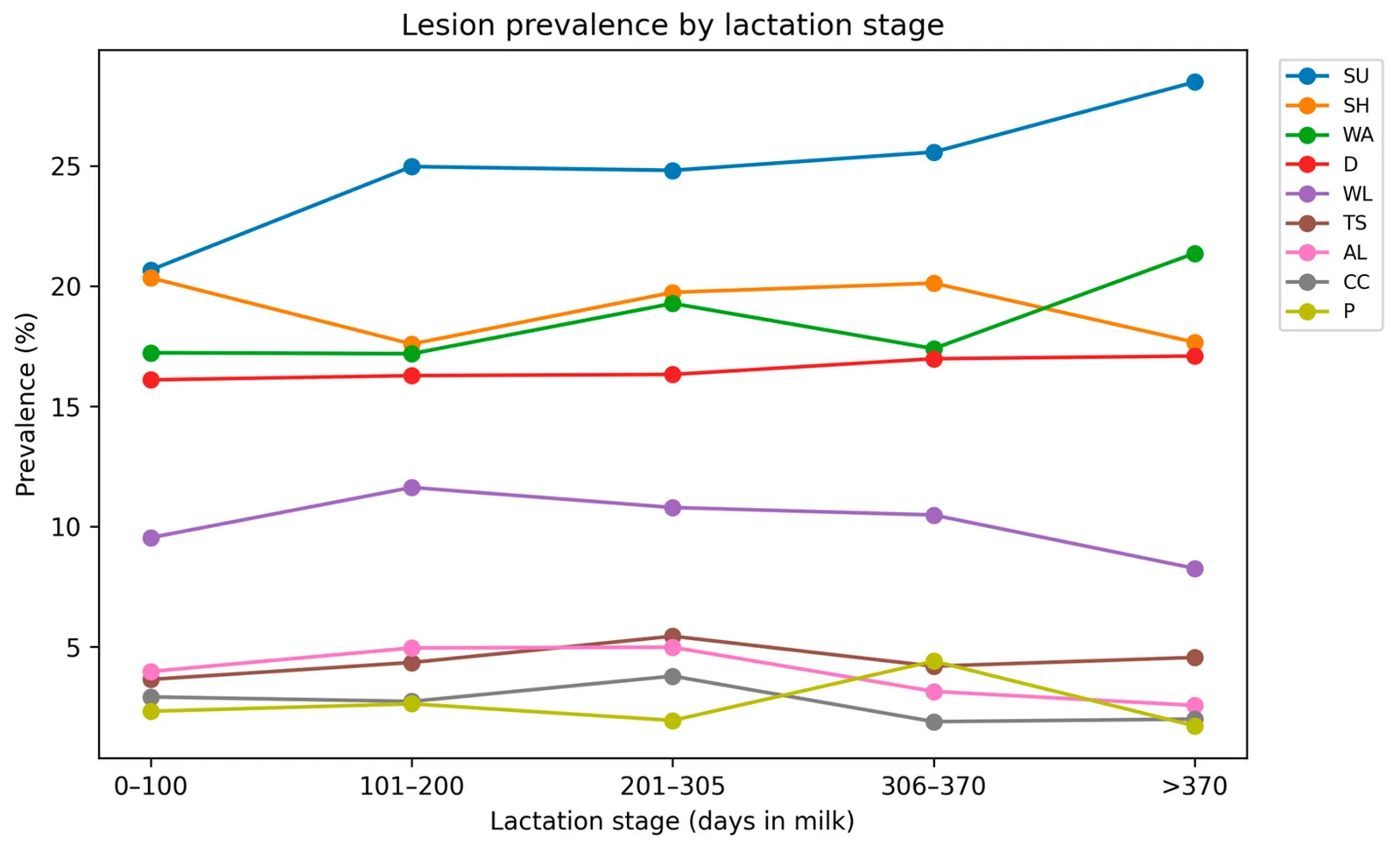
Prevalence of individual claw lesion types according to stage of lactation. Lactation stage was categorized as 0–100, 101–200, 201–305, 306–370, and >370 days in milk (DIM).

Seasonal associations also differed among lesion types. Lower odds in spring remained significant after FDR correction for SU (OR = 0.46, 95% CI 0.38–0.57), SH (OR = 0.59, 95% CI 0.48–0.72), WA (OR = 0.65, 95% CI 0.52–0.81), and WL (OR = 0.59, 95% CI 0.46–0.77). Lower odds in autumn remained significant for SU and SH, whereas dermatitis showed higher odds in winter (OR = 2.03, 95% CI 1.58–2.60) and thin sole showed lower odds (OR = 0.31, 95% CI 0.15–0.67). Associations with lactation stage were less consistent (Figure 6). After FDR correction, only SU showed significant associations with lactation stage, with higher odds at 101–200 DIM (OR = 1.34, 95% CI 1.09–1.63) and >370 DIM (OR = 1.54, 95% CI 1.13–2.09) compared with 0–100 DIM. Full results of the nine lesion-specific models, including FDR-adjusted *p*-values, are reported in Supplementary Table S6.

### 3.4. Exploratory Analysis of Cumulative Milk Yield

**Figure 7:**
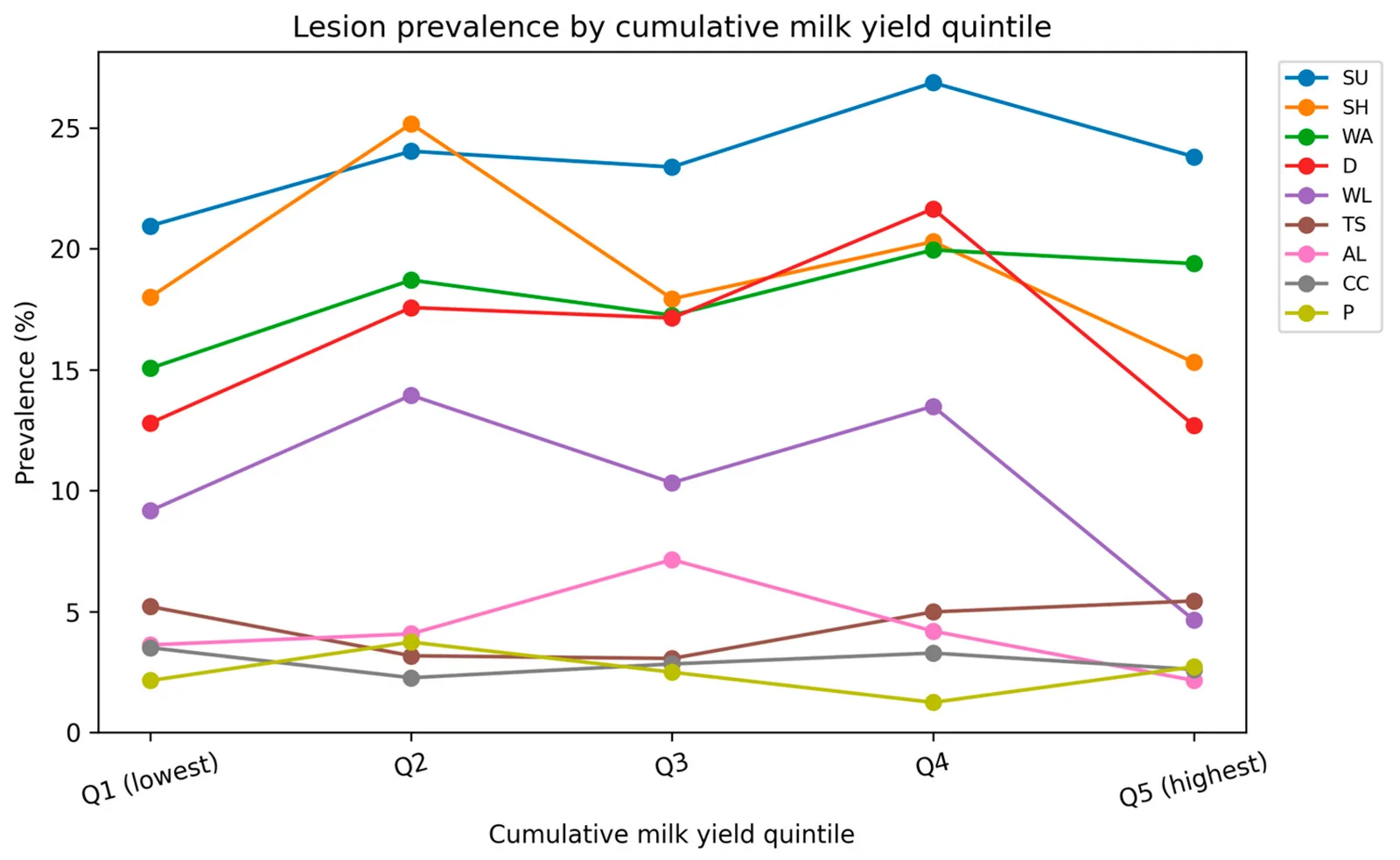
Prevalence of individual claw lesion types according to cumulative milk-yield quintile. The analysis is exploratory and does not account for DIM, parity, or repeated observations within cows.

In the exploratory analysis of cumulative milk yield, the prevalence of several lesion types varied across yield quintiles, generally increasing from the lower quintiles to the fourth quintile and then decreasing in the highest-yield group (Figure 7). Because this analysis did not account for DIM, parity, or repeated observations within cows, these patterns are presented descriptively and were not subjected to inferential testing.

### 3.5. Farm-Level Heterogeneity

**Figure 8:**
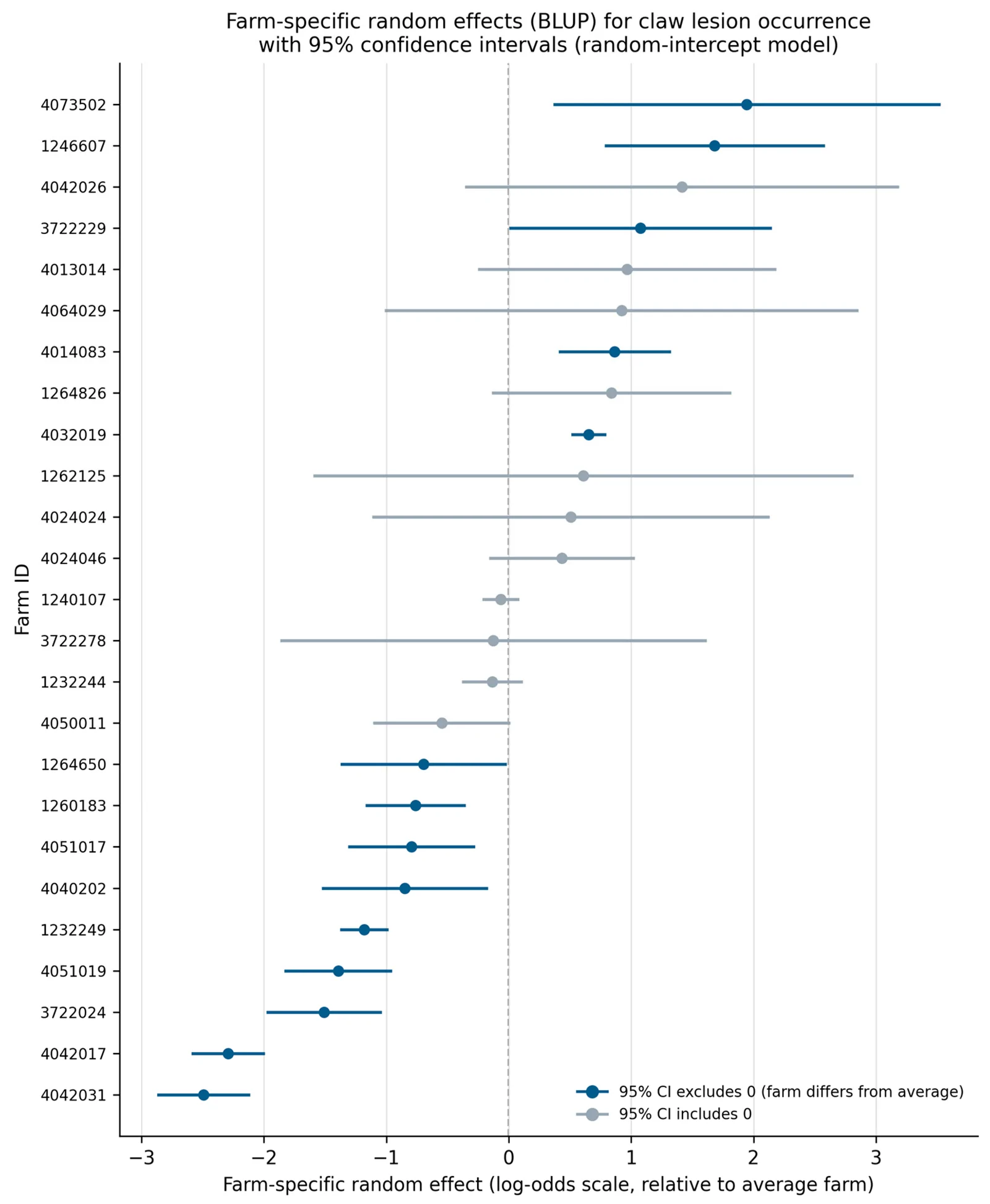
Caterpillar plot of farm-specific random effects (empirical Bayes estimates, log-odds scale) for the presence of at least one claw lesion, estimated from the farm random-intercept model. Farms are ordered by increasing random effect, and error bars represent 95% confidence intervals. Farm-specific estimates are shrunk toward the overall mean, with greater uncertainty for farms contributing fewer observations. The farm-level intraclass correlation coefficient was 0.347.

The estimates for parity changed little when farm was included in the model (Supplementary Table S7). For cows at parity ≥4, the OR was 3.12 (95% CI 2.48–3.91) in the original model, compared with 3.40 (95% CI 2.64–4.37) when farm was included as a fixed effect and 3.39 (95% CI 2.78–4.14) when it was included as a random intercept. Seasonal associations were less consistent after accounting for farm: autumn and winter were no longer significantly different from summer, while the lower odds observed in spring remained significant. The fixed- and random-effect models gave similar results overall. The random-intercept model also showed substantial variability between farms (ICC = 0.347), as shown by the farm-specific estimates in Figure 8. The number of visits varied considerably among farms, from 1 to 1251, and estimates were more uncertain for farms with fewer observations.

Given the potential contribution of observer-related differences to the variation observed across farms, we also examined the distribution of observations among trimmers. The dominant trimmer accounted for 80.5% of all visits and attended 22 of the 25 farms, while no farm was shared between trimmers. When the analysis was restricted to visits performed by this trimmer, considerable variation in raw lesion prevalence remained among farms (Supplementary Table S8), ranging from 12.2% to 100%, although the highest values were observed in farms with relatively few visits.

## 4. Discussion

This study characterized the prevalence, anatomical distribution, and risk factors for claw lesions in a population of 3066 Parmigiano Reggiano dairy cows across 25 farms, based on 4410 hoof trimming visits. Overall, 53.5% of visits and 44.3% of animals presented at least one claw lesion during the observation period, with sole ulcer, sole hemorrhage, and wall abscess the most frequently recorded conditions.

In-depth epidemiological studies on the Italian dairy cow population are still lacking, but a comparison with previous Italian studies about some specific cohort of animals shows considerable variation in both the overall frequency and the distribution of individual claw lesions. In a cross-sectional study including 3559 cows from 46 dairy herds in Northern Italy, Zecconi et al. reported digital dermatitis as the most frequent lesion (19.93%), followed by sole ulcer (10.82%) and white line separation (9.83%) [24]. In our population, sole ulcer was more frequent (17.1% of cows), whereas dermatitis was recorded in 12.0% of animals. More recently, Alrhmoun et al., studying 2223 cows from 131 Italian farms, reported prevalences of 18.85% for sole ulcer and 23.44% for digital dermatitis, while considerably higher values were found for sole hemorrhage (49.21%) and white line disease (43.9%) [25]. Although the prevalence of sole ulcer in that study was similar to that observed in our population, direct comparison should be made with caution because different breeds, geographical areas and recording protocols were included.

A useful comparison can also be made with the recent study of Capuzzello et al. on dairy farms in Southern Italy [26]. Over a four-year period, 64.1% of the 4329 cows examined had at least one lesion, compared with 44.3% in the present study. However, when individual examinations were considered, lesions were detected in 40.1% of inspections, compared with 53.5% of visits in our dataset. The longer follow-up and greater number of repeated examinations in the Southern Italian study may partly explain this difference.

Our population showed a higher prevalence of mechanically associated disorders, particularly SU, SH and WA, while dermatitis was less frequent than in other Italian studies. Whether this pattern is related to characteristics of the Parmigiano Reggiano production system cannot be established from the present data and would require a direct comparison with farms outside this production chain, but with similar characteristics.

Lesions of mechanical origin (SU, SH, WA, WL) were predominantly located on the hind lateral claws (positions 5-HRL and 8-HLL), as reported in the literature [5,26,27,28]. This is generally related to the different distribution of load between the two claws of the hind foot. The lateral claw is subjected to greater and more variable loading during standing and locomotion and is therefore more susceptible to horn disruption and sole lesions [29]. SU, SH, WA, and WL showed moderate pairwise co-occurrence within the same visit consistent with a shared mechanical/metabolic origin. Dermatitis was essentially uncorrelated with the other lesion types, supporting a distinct etiological pathway.

The predominance of infectious lesions (D and P) in the hind feet agrees with previous studies reporting that digital dermatitis occurs mainly on the plantar aspect of the hind limbs [14,30,31,32] and that infectious hoof lesions are generally more prevalent in rear than in front feet as exposure to excessive manure and moisture has been associated with an increased risk [14,16].

In the present study, parity/age was the strongest individual factor associated with lesion occurrence, while substantial variation was also observed among farms. Lactation stage showed weaker and less consistent associations. Compared with first-parity cows, the odds of having at least one lesion increased across the parity categories used in the model and were approximately three times higher in cows at parity ≥4. In the secondary lesion-specific analyses, the association with parity was particularly evident for SU, SH, and WA, and these associations remained significant after FDR correction. The pattern was less consistent for the other lesion types. Similar associations between increasing parity or age and claw lesions have been reported previously [3,6,33,34] and may reflect the cumulative effects of prolonged exposure to hard surfaces and mechanical trauma, together with changes in claw horn quality and the metabolic and hormonal changes occurring around calving [12]. A different pattern was observed for dermatitis, for which parity was not significantly associated with lesion occurrence in our population. Previous studies have reported a decreasing prevalence of digital dermatitis with increasing parity, possibly reflecting the development of acquired immunity following repeated exposure [5,30,35]. However, this pattern is not consistent across studies, for example, Oberbauer et al. reported an increased risk of digital dermatitis with increasing parity [34]. Associations with lactation stage were less consistent than those observed for parity. After FDR correction, only SU remained associated with lactation stage, and the pattern across DIM categories was not monotonic. These findings therefore do not support a clear stage-specific pattern and should be interpreted cautiously.

The parity ≥4 category was retained as a single group for the multivariable model to preserve statistical power, but this pooling should be interpreted with caution. When prevalence was examined at each individual parity value, it did not increase monotonically beyond parity 4: raw prevalence of at least one lesion rose from 40.6% (parity 1) to a peak of 73.9% at parity 5, then declined to 68.4% at parity 6, 62.5% at parity 7, and 50.0% at parity 8 (Table 4). However, the number of visits at parity ≥6 was small (*n* = 95, 32, and 4, respectively), yielding wide confidence intervals (e.g., 15.0–85.0% at parity 8) that preclude firm conclusions about a true decrease in lesion prevalence. This apparent reversal may reflect a biological resistance or may be partly explained by selective culling, as cows with severe claw disorders are less likely to remain in the herd for several lactations. Distinguishing between these two explanations was not possible with the present cross-sectional dataset and would require longitudinal culling data.

The exploratory analysis of cumulative milk yield showed a non-linear pattern, with lesion prevalence decreasing in the highest-yield quintile. However, cumulative milk yield is closely related to DIM and parity. In the literature, higher milk yield has been associated with an increased risk of lameness [8], while claw lesions may themselves reduce milk production; for example, sole ulcer and white line disease have been associated with losses of approximately 570 and 370 kg of milk per lactation, respectively [36]. The lower prevalence observed in the highest-yield group may therefore reflect reverse causation rather than a protective effect of higher milk production. A standardized or DIM- and parity-adjusted measure of milk yield would be needed to investigate this relationship further.

Season was also associated with lesion occurrence, with lower odds in spring and autumn and higher odds in winter compared with summer in the primary model. At the lesion-specific level, several seasonal associations remained significant after FDR correction, although their direction and magnitude differed among lesion types. Previous studies have also reported seasonal variation in claw lesions, including a winter increase in Southern Italian dairy herds [26], whereas Sanders et al. reported higher rates of several claw lesions during summer in the southeastern United States [37]. These differences may reflect variations in climate, housing and herd management. In the present study, however, the temporal pattern was less clear when examined in greater detail. Monthly prevalence varied considerably, from 30.2% in March to 73.6% in August, with substantial differences even between months within the same season. The number of visits was also unevenly distributed across months, ranging from 103 in January to 953 in September. Moreover, sensitivity analyses showed that some seasonal associations changed after accounting for calendar year: the higher odds observed in winter were no longer significant, and the difference between autumn and summer was not retained when the analysis was restricted to months represented in both years. Spring was the most consistent finding, with lower odds than summer across the different models. The month-based model also showed marked variation between individual months. These findings suggest that the temporal pattern observed in this dataset may reflect not only season, but also calendar year and the scheduling of hoof-trimming visits rather than a biological seasonal effect.

The random-intercept model showed substantial variability between farms in lesion occurrence (ICC = 0.347), even after accounting for individual cow factors. Some of this variation may also be related to differences in how hoof trimmers recognized and recorded lesions. Since farms were not shared among trimmers and inter-observer agreement was not formally assessed, it was not possible to fully separate farm and observer effects. However, lesion prevalence still varied considerably among the 22 farms attended by the dominant trimmer, suggesting that differences between trimmers are unlikely to account for all of the observed farm variation. Herd-level conditions may therefore also play an important role in claw health. Such variability is not unexpected, given that farms within the Parmigiano Reggiano system have been shown to differ substantially in structural and management characteristics—including herd size, housing type, feeding system, and stocking rate—even within the same geographically restricted production chain [38].

The electronic recording system used in the present study may have improved the efficiency and accuracy of hoof-health data collection by reducing transcription errors and facilitating standardized lesion recording [39]. In addition to simplifying data entry and reporting, structured digital records can support disease surveillance, benchmarking, risk factor assessment, and genetic improvement [16,21]. The integration of these data with productive and genetic databases could further increase their value for herd and population-level monitoring. However, Solano et al. report a low within-trimmer correlation of lesion observations in their study [5], so some observer-related bias cannot be excluded because lesion identification relies only on visual assessment.

### Study Limitations

Data collection included mainly scheduled routine trimming visits (*n* = 3937) together with 473 urgent or on-demand visits, generally performed when lameness or a claw disorder was already suspected. The inclusion of these visits could therefore have led to some overestimation of lesion prevalence. However, when the analysis was restricted to routine visits, prevalence decreased only from 53.5% to 50.1% at the visit level and from 44.3% to 42.0% at the animal level. The reported prevalence should be interpreted as referring to cows undergoing hoof trimming, with a small proportion of animals selected because of suspected claw problems, rather than to a randomly sampled population.

Lesions were recorded by different professional hoof trimmers, and inter-observer agreement was not formally assessed. Since each farm was visited by only one trimmer, farm and observer effects could not be completely separated. Nevertheless, considerable variation among farms was still present when the analysis was restricted to the dominant trimmer. Another limitation was the uneven number of observations per cow, as 73% of cows had only one recorded visit, which limited the use of more complex hierarchical models. Information on housing, flooring, hygiene, feeding, and other herd-level factors was also unavailable, so the reasons for the observed differences among farms could not be directly investigated. In addition, the 25 herds were not selected to be a representative sample of the entire Parmigiano Reggiano production system. For this reason the results should be generalized beyond the studied farms with caution.

## 5. Conclusions

Claw lesions were common in this population of Parmigiano Reggiano dairy cows, affecting 44.3% of animals and being recorded in 53.5% of visits. Sole ulcer, sole hemorrhage and wall abscess were the most frequent lesions. Parity/age showed the strongest association with lesion occurrence, particularly for mechanically associated lesions, although the independent contributions of parity and age could not be disentangled. Associations with lactation stage were weak. Differences among farms remained evident after adjustment for individual cow factors, suggesting that herd-level conditions may contribute to variation in claw health.

Systematic electronic recording by hoof trimmers can provide useful data for monitoring claw health, both within and across herds, although consistent lesion identification and recording are essential. Recorded claw-health data could complement existing national animal-health and welfare monitoring frameworks, such as ClassyFarm and the National Quality System for Animal Welfare (SQNBA), by providing standardized information on hoof health that could support herd-level monitoring and welfare assessment. Further studies should include information on housing, flooring, feeding and other management factors that could help explain the differences observed among farms. Comparison with dairy herds outside the Parmigiano Reggiano production system would also be useful to identify possible characteristics of this specific production context. Integration of standardized hoof-trimming records with national cattle databases could further extend their use to large-scale disease surveillance and, potentially, genetic evaluation of claw health.

## Figures and Tables

**Table 1:** Visit-level and animal-level prevalence of claw lesions recorded during hoof-trimming visits in 3066 dairy cows from 25 Parmigiano Reggiano herds. Lesion types are reported as the number and percentage of visits and animals with at least one occurrence during the study period.

Type of Lesion	N Visits	% Visits	N Animals	% Animals
SU—Sole ulcer	1050	23.8%	523	17.1%
SH—Hemorrhage	853	19.3%	430	14.0%
WA—Wall abscess	797	18.1%	429	14.0%
D—Dermatitis (incl. DD)	722	16.4%	369	12.0%
WL—Wall lesion	455	10.3%	217	7.1%
TS—Thin sole	193	4.4%	96	3.1%
AL—Apical lesion	187	4.2%	73	2.4%
CC—Corkscrew claw	128	2.9%	75	2.4%
P—Phlegmon	109	2.5%	59	1.9%
other_rare (T, HE, L)	45	1.0%	-	-

**Table 2:** Multivariable logistic regression analysis of cow-level factors associated with the presence of at least one claw lesion during hoof-trimming visits. Odds ratios (OR), 95% confidence intervals (CI), and *p*-values are reported for parity, stage of lactation, and season. Standard errors were clustered at the animal level to account for repeated visits within cows.

Predictor	OR	95% CI	*p*
Parity 2 (vs. 1)	1.41	1.15–1.71	0.001
Parity 3 (vs. 1)	2.52	2.01–3.15	<0.001
Parity 4+ (vs. 1)	3.14	2.50–3.94	<0.001
Lactation 101–200 d (vs. 0–100)	0.99	0.84–1.17	0.905
Lactation 201–305 d (vs. 0–100)	1.24	1.06–1.46	0.008
Lactation 306–370 d (vs. 0–100)	1.29	1.03–1.60	0.025
Lactation > 370 d (vs. 0–100)	1.23	0.95–1.59	0.120
Autumn (vs. Summer)	0.82	0.70–0.96	0.011
Winter (vs. Summer)	1.46	1.17–1.81	0.001
Spring (vs. Summer)	0.62	0.53–0.73	<0.001

**Table 3:** Monthly distribution of visits and lesion prevalence. Prevalence was calculated as the percentage of visits in which at least one claw lesion was recorded.

Month	Season	N Visits	N with Lesion	Prevalence (%)
January	Winter	103	71	68.9
February	Winter	108	71	65.7
March	Spring	341	103	30.2
April	Spring	338	164	48.5
May	Spring	452	238	52.7
June	Summer	285	199	69.8
July	Summer	667	278	41.7
August	Summer	406	299	73.6
September	Autumn	953	569	59.7
October	Autumn	325	101	31.1
November	Autumn	143	86	60.1
December	Winter	289	182	63.0

**Table 4:** Prevalence of at least one claw lesion by exact parity value. Wilson score 95% confidence intervals. Note the wide intervals at parity ≥6, reflecting small sample sizes (*n* = 95, 32, and 4 visits, respectively); the apparent decline beyond parity 5 should be interpreted with caution and may reflect survivor/selection bias rather than a true reduction in risk.

Parity	N Visits	N with Lesion	Prevalence (%)	95% CI
1	1410	572	40.6	38.0–43.2
2	1161	577	49.7	46.8–52.6
3	915	580	63.4	60.2–66.4
4	540	358	66.3	62.2–70.2
5	253	187	73.9	68.2–78.9
6	95	65	68.4	58.5–76.9
7	32	20	62.5	45.3–77.1
8	4	2	50.0	15.0–85.0

## Data Availability

The data supporting the findings of this study are not publicly available due to privacy and institutional restrictions but may be provided by the corresponding author upon reasonable request.

## Supplementary Materials

The following supporting information can be downloaded at: https://www.mdpi.com/article/10.3390/ani16203195/s1, Table S1: Workload distribution across hoof trimmers and raw lesion prevalence by trimmer; Table S2: Mapping of Datatrim lesion codes to the consolidated lesion categories used in the study and to ICAR Claw Health Atlas terminology [40]; Table S3: Multivariable model for the presence of at least one claw lesion with calendar year added to the main model; Table S4: Multivariable model with calendar month in place of season; Table S5: Multivariable model restricted to the five calendar months represented in both study years; Table S6: Lesion-specific multivariable logistic models with Benjamini–Hochberg FDR-adjusted p-values; Table S7: Sensitivity analysis of the main model with farm included as a fixed effect or as a random intercept; Table S8: Raw lesion prevalence by farm among visits performed by the dominant trimmer; Methods S1: Statistical software and model implementation details [41,42].
