# Supplementary material for: Hoof Health in the Parmigiano Reggiano Dairy Production System: A Pilot Epidemiological Study Using Digitally Recorded Hoof-Trimming Data
Source: Animals (Basel). 2026 Oct 12;16(20):3195. doi: 10.3390/ani16203195 (PMC13700555; doi:10.3390/ani16203195)
Supplement: Supplementary file 1 [file animals-16-03195-s001.zip › animals-4602845-supplementary.pdf]

Supplementary Table S1.
Workload distribution across the four hoof trimmers who contributed data to the study, including number of visits, animals and farms attended, share of total visits, and raw (unadjusted) lesion prevalence.

Trimmer	Visits (n)	Animals (n)	Farms (n)	% of total visits	Raw prevalence (%)
1	43	43	2	1.0%	39.5%
2	793	532	2	18.0%	45.6%
3	26	26	1	0.6%	84.6%
4	3,548	2,485	22	80.5%	55.0%

Supplementary Table S2.
Mapping of the original Datatrim lesion codes to the consolidated lesion categories used in the present study and to the corresponding standardized terminology of the ICAR Claw Health Atlas [40].

Datatrim	ICAR	ICAR Definition
Sole Ulcer (SU)	Sole Ulcer (SU)	Penetration through the sole horn exposing fresh or necrotic corium
Apical Lesion (AL)	Toe Ulcer (TU)<br>+<br>Toe Necrosis (TN)	Ulcer located at the toe<br><br>Necrosis of the tip of the toe with involvement of bone tissue
Sole Hemorrhage (SH)	Sole Hemorrhage (SH)	Diffused and/or circumscribed red or yellow discoloration of the sole and/or white line
Wall Abscess (WA)	White Line Abscess (WLA)	Necro-purulent inflammation of the corium
Wall Lesion (WL)	White Line Fissure (WLF)	Separation of the white line which remains after balancing both soles
Thin Sole (TS)	Thin Sole (TS)	Sole horn yields (feels spongy) when finger pressure is applied
Dermatitis (D)	Digital Dermatitis (DD)<br>+ Interdigital Dermatitis (ID)	Infection of the digital and/or interdigital skin with erosion, mostly painful ulcerations and/or chronic hyperkeratosis/proliferation<br>+<br>All kind of mild dermatitis around the claws, that is not classified as digital dermatitis

Datatrim	ICAR	ICAR Definition
Corkscrew claw (CC)	Corkscrew claw (CC)	Any torsion of either the outer or inner claw. The dorsal edge of the wall deviates from a straight line
Phlegmon (P)	Interdigital Phlegmon (IP)	Symmetric painful swelling of the foot commonly accompanied with odorous smell with sudden onset of lameness

Supplementary Table S3.
Multivariable model for the presence of at least one claw lesion, full dataset (n = 4,410), with calendar year added to the main model (parity, season, stage of lactation). Standard errors clustered at the animal level.

Term	OR	95% CI	p
Parity 2 vs 1	1.34	1.10–1.63	0.004
Parity 3 vs 1	2.45	1.96–3.07	<0.001
Parity 4+ vs 1	3.16	2.51–3.97	<0.001
Autumn vs Summer	0.75	0.64–0.87	<0.001
Winter vs Summer	1.09	0.87–1.37	0.451
Spring vs Summer	0.64	0.54–0.76	<0.001
DIM 101–200 vs 0–100	1.05	0.88–1.24	0.585
DIM 201–305	1.31	1.11–1.53	0.001
DIM 306–370	1.39	1.12–1.74	0.003
DIM >370	1.34	1.03–1.74	0.031
Year 2024 vs 2023	0.54	0.45–0.64	<0.001

Supplementary Table S4.
Multivariable model with exact calendar month in place of season (reference: July), keeping parity and lactation stage (DIM) as in the main model, full dataset (n = 4,410). AIC = 5,587.8, versus 5,592.7 for the equivalent month-based model without DIM, and 5,814.9 for the season-based model without year. Standard errors clustered at the animal level.

Term	OR	95% CI	p
Parity 2 vs 1	1.33	1.08–1.63	0.007
Parity 3 vs 1	2.53	2.01–3.19	<0.001
Parity 4+ vs 1	3.00	2.37–3.78	<0.001
DIM 101–200 vs 0–100	1.08	0.91–1.28	0.385
DIM 201–305	1.26	1.07–1.48	0.007

Term	OR	95% CI	p
DIM 306–370	1.40	1.11–1.75	0.004
DIM >370	1.24	0.95–1.63	0.117
January vs July	3.48	2.24–5.42	<0.001
February vs July	2.80	1.85–4.25	<0.001
March vs July	0.67	0.50–0.89	0.006
April vs July	1.30	0.99–1.69	0.055
May vs July	1.64	1.28–2.12	<0.001
June vs July	3.19	2.35–4.32	<0.001
August vs July	4.42	3.35–5.85	<0.001
September vs July	2.02	1.63–2.49	<0.001
October vs July	0.68	0.51–0.90	0.008
November vs July	2.11	1.48–3.02	<0.001
December vs July	2.59	1.93–3.47	<0.001

Supplementary Table S5.

Multivariable model (parity, stage of lactation, season, and calendar year) restricted to the five calendar months represented in both study years (March, July, August, September, and October; n = 2,692 visits from 2,137 cows). Standard errors were clustered at the animal level.

Term	OR	95% CI	p
Parity 2 vs 1	1.26	1.00–1.59	0.053
Parity 3 vs 1	1.96	1.52–2.54	<0.001
Parity 4+ vs 1	2.54	1.94–3.32	<0.001
Autumn vs Summer	0.86	0.73–1.02	0.089
Spring vs Summer	0.40	0.31–0.53	<0.001
DIM 101–200 vs 0–100	1.03	0.81–1.30	0.816
DIM 201–305	1.39	1.11–1.74	0.004
DIM 306–370	1.54	1.17–2.03	0.002
DIM >370	1.52	1.08–2.13	0.015
Year 2024 vs 2023	0.33	0.26–0.42	<0.001

Supplementary Table S6.

Full results of the nine lesion-specific multivariable logistic models (exploratory analyses), with odds ratios (OR), 95% confidence intervals (CI), nominal p-values, and p-values adjusted for multiple testing across all 90 coefficients using the Benjamini-Hochberg false discovery rate (FDR) procedure. Reference categories: parity 1, summer, 0-100 days in milk (DIM). Standard errors clustered at the animal level. 18 of 90 coefficients yellow remained statistically significant after FDR correction (q < 0.05).

Lesion	Term	OR	95% CI	p	q (FDR)
SU	Parity 2 vs 1	1.61	1.19–2.19	0.002	0.016
SU	Parity 3 vs 1	3.16	2.29–4.36	<0.001	<0.001
SU	Parity 4+ vs 1	3.80	2.77–5.22	<0.001	<0.001

Lesion	Term	OR	95% CI	p	q (FDR)
SU	Spring vs Summer	0.46	0.38–0.57	<0.001	<0.001
SU	Autumn vs Summer	0.77	0.65–0.92	0.003	0.020
SU	Winter vs Summer	1.13	0.89–1.43	0.306	0.530
SU	DIM 101-200 vs 0-100	1.34	1.09–1.63	0.005	0.027
SU	DIM 201-305 vs 0-100	1.30	1.06–1.58	0.011	0.050
SU	DIM 306-370 vs 0-100	1.26	0.96–1.64	0.091	0.256
SU	DIM >370 vs 0-100	1.54	1.13–2.09	0.006	0.032
SH	Parity 2 vs 1	1.20	0.87–1.64	0.261	0.509
SH	Parity 3 vs 1	1.89	1.36–2.63	<0.001	0.001
SH	Parity 4+ vs 1	2.55	1.84–3.53	<0.001	<0.001
SH	Spring vs Summer	0.59	0.48–0.72	<0.001	<0.001
SH	Autumn vs Summer	0.68	0.56–0.81	<0.001	<0.001
SH	Winter vs Summer	0.88	0.68–1.13	0.306	0.530
SH	DIM 101-200 vs 0-100	0.84	0.69–1.04	0.105	0.263
SH	DIM 201-305 vs 0-100	0.96	0.79–1.16	0.654	0.774
SH	DIM 306-370 vs 0-100	0.95	0.74–1.23	0.693	0.791
SH	DIM >370 vs 0-100	0.83	0.60–1.15	0.271	0.509
WA	Parity 2 vs 1	1.24	0.92–1.67	0.167	0.366
WA	Parity 3 vs 1	2.00	1.44–2.78	<0.001	<0.001
WA	Parity 4+ vs 1	2.19	1.58–3.04	<0.001	<0.001
WA	Spring vs Summer	0.65	0.52–0.81	<0.001	<0.001
WA	Autumn vs Summer	0.97	0.80–1.17	0.759	0.833
WA	Winter vs Summer	1.29	1.00–1.67	0.052	0.168
WA	DIM 101-200 vs 0-100	1.02	0.82–1.28	0.830	0.869
WA	DIM 201-305 vs 0-100	1.18	0.95–1.46	0.139	0.313
WA	DIM 306-370 vs 0-100	0.99	0.75–1.31	0.926	0.937
WA	DIM >370 vs 0-100	1.31	0.93–1.83	0.121	0.295
D	Parity 2 vs 1	1.32	0.97–1.78	0.074	0.221
D	Parity 3 vs 1	1.67	1.19–2.35	0.003	0.020
D	Parity 4+ vs 1	1.38	0.95–2.01	0.094	0.256
D	Spring vs Summer	0.93	0.76–1.14	0.505	0.668
D	Autumn vs Summer	0.94	0.77–1.14	0.540	0.675
D	Winter vs Summer	2.03	1.58–2.60	<0.001	<0.001
D	DIM 101-200 vs 0-100	1.04	0.85–1.28	0.691	0.791
D	DIM 201-305 vs 0-100	1.08	0.88–1.32	0.466	0.658
D	DIM 306-370 vs 0-100	1.09	0.83–1.43	0.528	0.675
D	DIM >370 vs 0-100	1.11	0.79–1.55	0.552	0.681
WL	Parity 2 vs 1	1.12	0.75–1.68	0.574	0.689
WL	Parity 3 vs 1	1.56	1.00–2.44	0.052	0.168
WL	Parity 4+ vs 1	1.69	1.07–2.67	0.023	0.095
WL	Spring vs Summer	0.59	0.46–0.77	<0.001	<0.001
WL	Autumn vs Summer	0.78	0.62–0.97	0.026	0.102
WL	Winter vs Summer	0.88	0.62–1.25	0.480	0.658
WL	DIM 101-200 vs 0-100	1.28	0.95–1.71	0.102	0.262
WL	DIM 201-305 vs 0-100	1.15	0.88–1.51	0.292	0.530

Lesion	Term	OR	95% CI	p	q (FDR)
WL	DIM 306-370 vs 0-100	1.07	0.76–1.52	0.694	0.791
WL	DIM >370 vs 0-100	0.85	0.56–1.29	0.446	0.658
TS	Parity 2 vs 1	0.83	0.46–1.49	0.531	0.675
TS	Parity 3 vs 1	1.27	0.67–2.40	0.470	0.658
TS	Parity 4+ vs 1	1.99	1.07–3.70	0.029	0.108
TS	Spring vs Summer	0.62	0.42–0.93	0.021	0.089
TS	Autumn vs Summer	0.79	0.57–1.08	0.139	0.313
TS	Winter vs Summer	0.31	0.15–0.67	0.003	0.019
TS	DIM 101-200 vs 0-100	1.20	0.74–1.95	0.459	0.658
TS	DIM 201-305 vs 0-100	1.45	0.93–2.24	0.099	0.262
TS	DIM 306-370 vs 0-100	1.09	0.62–1.89	0.774	0.839
TS	DIM >370 vs 0-100	1.23	0.61–2.46	0.565	0.687
AL	Parity 2 vs 1	1.27	0.64–2.50	0.489	0.658
AL	Parity 3 vs 1	2.21	1.07–4.54	0.031	0.113
AL	Parity 4+ vs 1	1.92	0.93–3.97	0.078	0.226
AL	Spring vs Summer	0.77	0.50–1.20	0.247	0.503
AL	Autumn vs Summer	0.96	0.69–1.34	0.803	0.850
AL	Winter vs Summer	1.09	0.64–1.86	0.754	0.833
AL	DIM 101-200 vs 0-100	1.26	0.80–1.98	0.314	0.533
AL	DIM 201-305 vs 0-100	1.27	0.81–2.00	0.297	0.530
AL	DIM 306-370 vs 0-100	0.75	0.39–1.45	0.393	0.632
AL	DIM >370 vs 0-100	0.62	0.28–1.33	0.219	0.459
CC	Parity 2 vs 1	0.99	0.51–1.91	0.982	0.982
CC	Parity 3 vs 1	1.44	0.68–3.03	0.340	0.566
CC	Parity 4+ vs 1	1.76	0.86–3.63	0.125	0.295
CC	Spring vs Summer	0.77	0.49–1.22	0.270	0.509
CC	Autumn vs Summer	0.97	0.64–1.46	0.866	0.896
CC	Winter vs Summer	0.82	0.43–1.54	0.538	0.675
CC	DIM 101-200 vs 0-100	0.94	0.58–1.51	0.791	0.847
CC	DIM 201-305 vs 0-100	1.29	0.83–2.00	0.252	0.503
CC	DIM 306-370 vs 0-100	0.62	0.29–1.33	0.219	0.459
CC	DIM >370 vs 0-100	0.66	0.22–1.99	0.465	0.658
P	Parity 2 vs 1	1.30	0.67–2.54	0.434	0.658
P	Parity 3 vs 1	1.04	0.47–2.31	0.919	0.937
P	Parity 4+ vs 1	0.38	0.13–1.10	0.074	0.221
P	Spring vs Summer	0.84	0.55–1.29	0.418	0.658
P	Autumn vs Summer	0.64	0.41–0.99	0.045	0.155
P	Winter vs Summer	0.65	0.24–1.74	0.389	0.632
P	DIM 101-200 vs 0-100	1.12	0.61–2.04	0.721	0.812
P	DIM 201-305 vs 0-100	0.82	0.49–1.37	0.442	0.658
P	DIM 306-370 vs 0-100	1.88	1.11–3.17	0.019	0.084
P	DIM >370 vs 0-100	0.72	0.28–1.85	0.490	0.658

Supplementary Table S7.
Sensitivity analysis for the multivariable model of the presence of at least one claw lesion, comparing odds ratios (OR) and 95% confidence intervals (CI) without farm adjustment, with farm included as a fixed effect (24 indicator variables), and with farm included as a random intercept (fitted by maximum likelihood via adaptive Gauss-Hermite quadrature; intraclass correlation coefficient = 0.347). Reference categories: parity 1, summer. Standard errors in the models were clustered at the animal level.

Term	No farm adjustment OR (95% CI)	Farm as fixed effect OR (95% CI)	Farm as random intercept OR (95% CI)
Parity 2 vs 1	1.38 (1.13–1.68)	1.34 (1.08–1.67)	1.35 (1.13–1.61)
Parity 3 vs 1	2.49 (1.99–3.12)	2.69 (2.10–3.45)	2.68 (2.20–3.27)
Parity 4+ vs 1	3.12 (2.48–3.91)	3.40 (2.64–4.37)	3.39 (2.78–4.14)
Spring vs Summer	0.61 (0.52–0.72)	0.68 (0.56–0.83)	0.68 (0.55–0.83)
Autumn vs Summer	0.82 (0.70–0.96)	1.10 (0.89–1.35)	1.10 (0.89–1.35)
Winter vs Summer	1.44 (1.16–1.79)	1.15 (0.90–1.48)	1.15 (0.88–1.50)

Supplementary Table S8.
Raw lesion prevalence by farm, restricted to visits performed by the dominant trimmer (Trimmer 4, 80.5% of all visits, 22 farms), sorted by increasing prevalence. Marked between-farm variability persists even within visits performed by a single observer, indicating that farm-level heterogeneity is not attributable solely to inter-trimmer differences.

Farm ID	Visits (n)	Raw prevalence (%)
4042017	394	12.2%
4042031	251	12.7%
4051019	99	31.3%
3722024	85	31.8%
4040202	38	34.2%
4050011	55	38.2%
1264650	34	47.1%
4051017	61	47.5%
1260183	101	49.5%
1240107	909	58.6%
4024046	49	63.3%
4032019	1237	74.5%
3722278	4	75.0%
4014083	120	80.0%
4024024	5	80.0%
3722229	20	85.0%
4013014	15	86.7%
1246607	47	91.5%
4042026	5	100.0%
1262125	1	100.0%
4064029	5	100.0%
4073502	13	100.0%

Supplementary Methods S1
Statistical software and model implementation details

S1.1. Software environment
All statistical analyses were performed in Python (version 3.11.15). Data handling was performed using pandas (version 3.0.2), numerical computations using NumPy (version 2.4.4), statistical and optimization functions using SciPy (version 1.17.1), and graphical outputs using Matplotlib (version 3.10.9). Logistic regression and mixed-effects models were fitted using custom functions based on NumPy and SciPy rather than pre-built statistical modelling packages.

S1.2. Multivariable logistic regression and cluster-robust standard errors
The primary analysis evaluated the association of parity, lactation stage, and season with the presence of at least one claw lesion during a hoof-trimming visit. The following multivariable logistic regression model was fitted:

$$logit[P(lesion = 1)] = \beta_0 + \beta_1(Parity\ 2) + \beta_2(Parity\ 3) + \beta_3(Parity \geq 4) + \beta_4(DIM\ 101\text{–}200) + \beta_5(DIM\ 201\text{–}305) + \beta_6(DIM\ 306\text{–}370) + \beta_7(DIM > 370) + \beta_8(Autumn) + \beta_9(Winter) + \beta_{10}(Spring)$$

Parity 1, 0–100 days in milk (DIM), and summer were used as the respective reference categories, with categorical predictors entered using indicator coding. The same model specification was subsequently fitted separately for each of the nine individual lesion types as secondary exploratory analyses.

Regression coefficients were estimated by maximum likelihood using Newton–Raphson iteration, equivalent to iteratively reweighted least squares (IRLS). At each iteration, fitted probabilities were calculated from the current coefficient estimates and the coefficients were updated using the corresponding score vector and information matrix. Iteration continued until the maximum absolute change in any coefficient was $<1 \times 10^{-10}$.

Because individual cows could contribute more than one hoof-trimming visit, standard errors were adjusted for within-animal clustering using a cluster-robust Huber–White sandwich variance estimator, with animal as the clustering unit. A finite-sample correction factor was applied:

$$c = [G/(G - 1)] \times [(N - 1)/(N - k)],$$

where G is the number of animal clusters, N is the number of observations, and k is the number of estimated model parameters. Odds ratios (ORs) were obtained by exponentiating the regression coefficients. Wald 95% confidence intervals were calculated on the log-odds scale using the cluster-robust standard errors and subsequently exponentiated. The finite-sample correction follows the approach described by Cameron and Miller [41].

S1.3. Farm-level sensitivity analyses and random-intercept model
To assess whether the associations observed in the primary model were affected by farm-level clustering, the model was refitted using two alternative approaches. First, farm was included as a fixed effect using

24 indicator variables, with one farm used as the reference category. Second, a generalized linear mixed model with a farm-level random intercept was fitted:

$$logit[P(lesion = 1)] = X\beta + u_j, \quad u_j \sim N(0, \sigma^2),$$

where u_j represents the random intercept for farm j. The fixed-effects specification for parity, lactation stage, and season was the same as in the primary model.

The random-intercept model was fitted by maximum marginal likelihood. The farm-specific random effect was integrated out using adaptive Gauss–Hermite quadrature with 11 quadrature nodes. For each trial value of σ, the mode of the penalized farm-specific log-likelihood was obtained by Newton iteration, and the quadrature nodes were recentered and rescaled around this mode using the local curvature.

Fixed-effect coefficients and the random-intercept variance were estimated jointly by maximizing the total marginal log-likelihood using the Nelder–Mead algorithm, with σ optimized on the logarithmic scale. Standard errors were obtained from the numerical Hessian of the negative log-likelihood using central finite differences.

The farm-level intraclass correlation coefficient (ICC) was calculated on the logistic latent-variable scale as:

$$ICC = \sigma^2/(\sigma^2 + \pi^2/3).$$

Farm-specific random effects were estimated as empirical Bayes posterior modes at the final fitted values of β and σ and were used to describe between-farm heterogeneity. Their uncertainty was estimated from the local curvature of the farm-specific penalized log-likelihood.

S1.4. Temporal sensitivity analyses

Additional sensitivity analyses were performed to examine whether the associations observed in the primary model were affected by the incomplete temporal coverage of the study period.
First, calendar year (2023 vs. 2024) was added as an additional categorical predictor to the primary model while retaining parity, lactation stage, and season.

Second, season was replaced by calendar month while retaining parity and lactation stage as covariates; July was used as the reference month. This analysis was used to assess temporal variation at a finer scale than the four-category seasonal classification.

Third, the primary model was repeated after restricting the dataset to the five calendar months represented in both study years (March, July, August, September, and October), with calendar year included as an additional covariate.

Finally, a further sensitivity analysis was performed using visits recorded between March and October, again including calendar year as an additional covariate.

All temporal sensitivity analyses were fitted using the same maximum-likelihood logistic regression procedure and animal-level cluster-robust variance estimator described in Section S1.2.

S1.5. Multiple-testing correction for lesion-specific analyses

The nine lesion-specific models were considered secondary exploratory analyses. Each model contained the same 10 non-intercept coefficients corresponding to parity, lactation stage, and season, resulting in 90 coefficient-level statistical tests.

To account for multiple testing, nominal p-values from these 90 coefficients were adjusted jointly using the Benjamini–Hochberg false discovery rate (FDR) procedure [42]. The 90 nominal p-values were ranked in ascending order, and initial adjusted values were calculated as:

$$p(i) \times 90/i,$$

where i represents the rank of the nominal p-value. Monotonicity of the adjusted values was enforced using a cumulative minimum from the largest to the smallest rank, and values were constrained to a maximum of 1. The resulting FDR-adjusted p-values are reported as q-values.

Associations with $q < 0.05$ were considered statistically significant in the lesion-specific analyses. Odds ratios and 95% confidence intervals were retained as the principal measures of the magnitude and precision of the estimated associations. Complete nominal and FDR-adjusted results are reported in Supplementary Table S6.
